# Structural and dynamic basis of substrate permissiveness in hydroxycinnamoyltransferase (HCT)

**DOI:** 10.1371/journal.pcbi.1006511

**Published:** 2018-10-26

**Authors:** Ying-Chih Chiang, Olesya Levsh, Chun Kei Lam, Jing-Ke Weng, Yi Wang

**Affiliations:** 1 Department of Physics, The Chinese University of Hong Kong, Shatin, Hong Kong; 2 Whitehead Institute for Biomedical Research, Cambridge, Massachusetts, United States of America; 3 Department of Biology, Massachusetts Institute of Technology, Cambridge, Massachusetts, United States of America; University of Uppsala, SWEDEN

## Abstract

Substrate permissiveness has long been regarded as the raw materials for the evolution of new enzymatic functions. In land plants, hydroxycinnamoyltransferase (HCT) is an essential enzyme of the phenylpropanoid metabolism. Although essential enzymes are normally associated with high substrate specificity, HCT can utilize a variety of non-native substrates. To examine the structural and dynamic basis of substrate permissiveness in this enzyme, we report the crystal structure of HCT from Selaginella moellendorffii and molecular dynamics (MD) simulations performed on five orthologous HCTs from several major lineages of land plants. Through altogether 17-*μ*s MD simulations, we demonstrate the prevalent swing motion of an arginine handle on a submicrosecond timescale across all five HCTs, which plays a key role in native substrate recognition by these intrinsically promiscuous enzymes. Our simulations further reveal how a non-native substrate of HCT engages a binding site different from that of the native substrate and diffuses to reach the catalytic center and its co-substrate. By numerically solving the Smoluchowski equation, we show that the presence of such an alternative binding site, even when it is distant from the catalytic center, always increases the reaction rate of a given substrate. However, this increase is only significant for enzyme-substrate reactions heavily influenced by diffusion. In these cases, binding non-native substrates ‘off-center’ provides an effective rationale to develop substrate permissiveness while maintaining the native functions of promiscuous enzymes.

## Author summary

Examples abound of enzymes that can process substrates other than their native ones. However, the structural and dynamic basis of this promiscuity remains to be fully understood. In this work, we examine HCT, an intrinsically promiscuous acyltransferase with conserved function in all land plants. We uncover the sub-microsecond swing motion of a key arginine residue facilitating the recognition of both native and non-native substrates of HCT. We also quantify the impact of an off-center binding site on the non-native reaction rate. Although our calculations were inspired by HCT, the results apply in general, *i.e*., for enzymes heavily influenced by diffusion, binding non-native substrates ‘off-center’, even with rather weak affinity, can accelerate non-native reactions to appreciable levels.

## Introduction

How enzymes evolve to acquire novel functions has attracted numerous studies on the subject of enzyme promiscuity, which can be subcategorized as substrate permissiveness, mechanistic elasticity, and concomitant product diversity [1–4]. The ability to recruit a non-native substrate may lead to the development of new enzymatic functions, or neofunctionalization, through rounds of mutation and selection. This process is generally thought to occur due to the presence and drift of intrinsic substrate/product permissiveness in the progenitor enzyme without significantly affecting its native function [5]. In plants, enzymes involved in specialized metabolism likely evolve from their primary counterparts through exploiting ancestral promiscuity [6, 7]. This makes plant specialized metabolism an ideal system to study the role of promiscuity in enzyme evolution. One interesting example is the hydroxycinnamoyl-CoA:shikimate hydroxycinnamoyltransferase (HCT), which belongs to the BAHD acyltransferase family involved in the biosynthesis of a diversity of ester- and amide-containing natural products [8]. HCT produces *p*-coumaroylshikimate (S1 Fig) by transferring the *p*-coumaroyl group from the acyl donor *p*-coumaroyl-CoA to the acyl acceptor shikimate. It is an essential enzyme in the phenylpropanoid metabolism, conserved across all land plants [9]. Interestingly, unlike many other essential metabolic enzymes, HCT exhibits relatively low affinity toward its native substrate shikimate and can utilize a variety of non-native substrates.

Previously, we crystallized the *A. thaliana* HCT (AtHCT) in the apo, *p*-coumaroyl-CoA-bound, and *p*-coumaroylshikimate-bound forms, as well as the *C. blumei* HCT (CbHCT) in complex with *p*-coumaroyl-CoA and the non-native acyl acceptor substrate 3-hydroxyacetophenone (3-HAP, see S1 Fig) [10]. Comparative analysis of these structures and multiple copies of 100-ns molecular dynamics (MD) simulations reveals that a conserved arginine acts as a ‘catalytic handle’—the residue adopts a primarily external conformation in the apo state of the enzyme, and swings to an internal conformation in the presence of the native substrate shikimate. In contrast, binding of the neutral, non-native substrate 3-HAP is unable to elicit such a response. This difference in active-site dynamics helps the promiscuous HCT to maintain the competitiveness of its native reaction over alternative non-native reactions. However, our previous MD simulations did not capture the transition between the external and internal states of the arginine handle, leaving the timescale of the transition and its mechanistic relevance to the enzymatic function an open question. It is also unknown whether such active site dynamics are a universal feature among HCTs. Furthermore, it remains a mystery how the non-native substrate 3-HAP, which binds at a site over 8 Å away from the catalytic center, engages its co-substrate *p*-coumaroyl-CoA as well as the catalytic residues of the HCT active site.

Here, in order to address the above questions, we report microsecond-long simulations performed on the specialized machine Anton [11] of HCTs from five land plants, namely, AtHCT, CbHCT, *Coffea canephora* HCT (CcHCT), *Sorghum bicolor* HCT (SbHCT), as well as the newly crystallized HCT from *Selaginella moellendorffii* (SmHCT). Through collectively 17-*μ*s simulations, we demonstrate the prevalent, sub-microsecond swing motion of the arginine handle across all five HCTs, and reveal how the non-native substrate 3-HAP engages its co-substrate and catalytic residues of the enzyme. Finally, by solving the Smoluchowski equation first for HCT and then for a generic enzyme model, we quantify the impact of off-center binding on 3-HAP reaction rate and examine in general how such binding facilitates non-native reactions in promiscuous enzymes.

## Results

### Extremely flexible arginine handle in SmHCT

**Fig 1:**
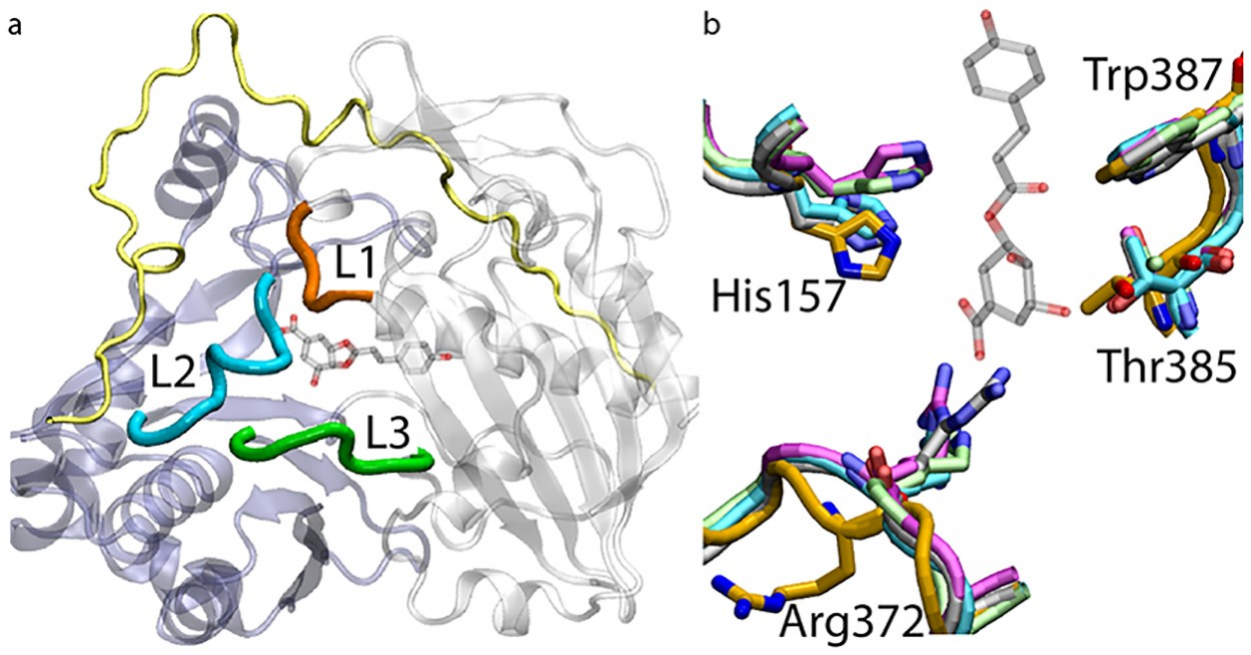
Crystal structure of apo SmHCT and comparison with other HCTs. (a) The two pseudosymmetric domains and the intervening loop in SmHCT are colored in blue, white and yellow, respectively, with three short loops (L1—L3) surrounding its active site entrance highlighted. (b) SmHCT residues are colored in gold, with AtHCT, CbHCT, CcHCT and SbHCT colored in lime, cyan, gray and magenta, respectively. Residues are numbered according to SmHCT (see S1 Table). For reference, the native product *p*-coumaroylshikimate from the holo AtHCT crystal structure is shown in both (a) and (b).

We crystallized the HCT ortholog from the lycophyte *Selaginella moellendorffii* and solved its apo structure at 2.9-Å resolution. The SmHCT crystal had a space group of P212121, and contained two molecules in the asymmetric unit. Several surface loops of the protein (residues 47-54, 216-237, and 259-262) have low electron density support and are omitted from the model. Similar to other HCT orthologs, the structure of SmHCT consists of two pseudo-symmetric domains (Fig 1a): one comprised of residues 1-179 and 392-411, the other of 241-391 and 412-451, and a long intervening loop consisting of residues 180-240. The SmHCT active site is located at the interface between its two domains with a cavity volume of 1372.2±10.4 Å^3^. Inside the active site, the universally conserved catalytic His157 acts as a general base to deprotonate the 5-hydroxyl of shikimate, priming it for the subsequent nucleophilic attack on *p*-coumaroyl-CoA. Apart from His157, two other key residues involved in substrate binding and catalysis, Thr385 and Trp387, are also structurally conserved in SmHCT compared to other available HCT structures (Fig 1b). However, there is a clear structural difference in the arginine handle, Arg372, which resides on one of the three short loops (L1) surrounding the entrance of the active site and coordinates the shikimate carboxylate with two salt bridges throughout the catalytic cycle. A lack of electron density for its side chain suggests that this residue is extremely flexible in SmHCT, occupying many different conformations in the apo state. This is in clear contrast to other crystallized HCT orthologs, which have defined density for the arginine handle. Since there is density to support the protein backbone in this region, Arg372 was modeled based on the location of its C_*α*_ atom and highest-probability side chain rotamer. We note that despite the ambiguity in its side chain conformation, the backbone of Arg372 already indicates that the residue protrudes away from the active site much more severely in SmHCT than in other known HCT orthologs (Fig 1b).

### Submicrosecond swing motion of the arginine handle across five HCTs

**Fig 2:**
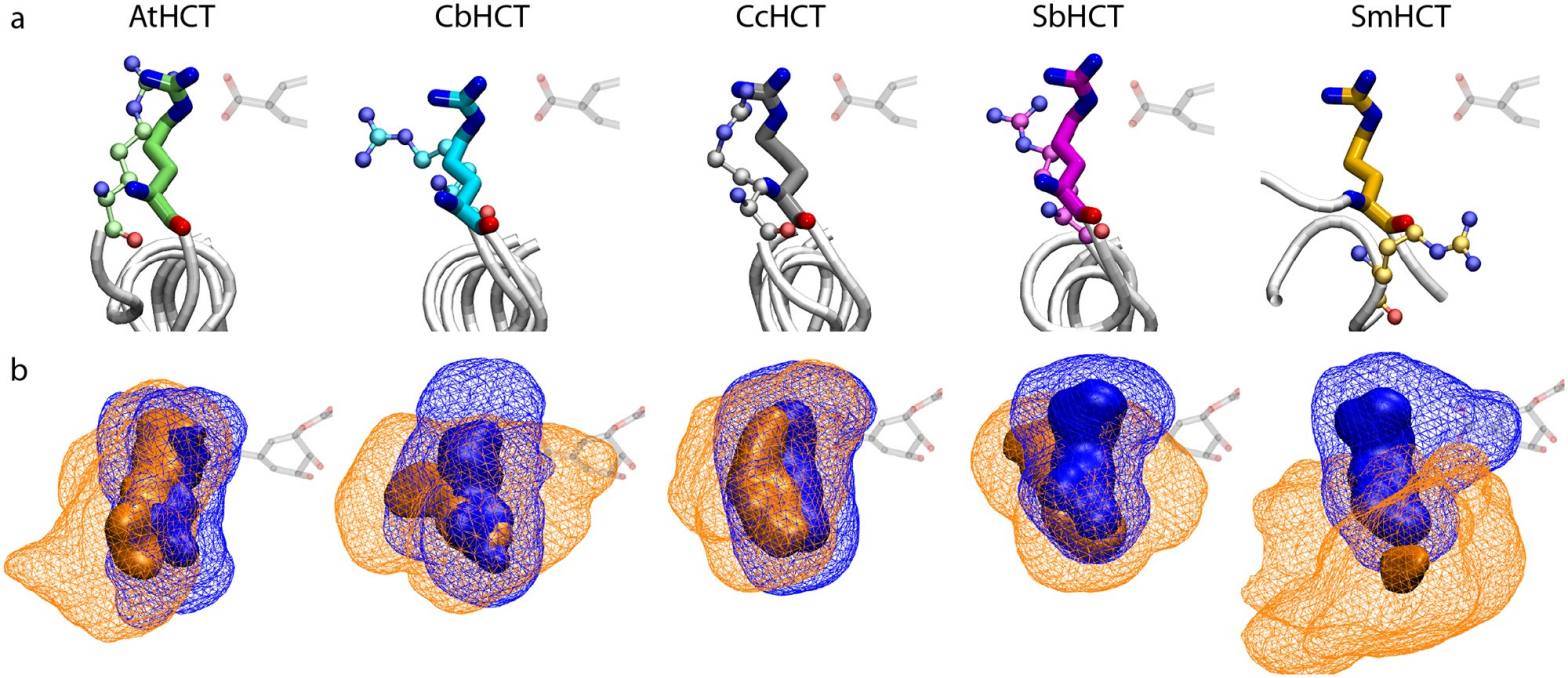
Conformation of the arginine handle in apo *vs*. holo HCT simulations. (a) The largest cluster from clustering analysis of the arginine handle in apo (thin sticks and balls) and holo (thick sticks) HCT simulations. (b) 3-D occupancy map of the arginine handle in apo (orange) and holo (blue) HCT simulations. The wireframe and solid meshes represent the isosurfaces of 1% and 50% occupancy, respectively. For reference, part of the product *p*-coumaroylshikimate from holo AtHCT crystal structure is shown in transparent.

In order to systematically examine their active-site dynamics, we performed microsecond-long simulations for five orthologous HCTs. Each HCT is simulated in both apo and holo states, with the latter containing the protein in complex with shikimate and *p*-coumaroyl-CoA (S2 Fig). For HCTs without holo crystal structures, their corresponding apo structures were used, with *p*-coumaroyl-CoA and shikimate manually introduced into the active site. To fully relax residues around the newly added substrates, a series of simulated annealing (SA) simulations were performed before the holo state Anton simulations were launched (see SI for details). These SA simulations can be considered as enforcing a rapid transition from the apo to holo state, while the subsequent Anton runs are used to collect statistics for the holo state. Consistent with our previous work [10], the Anton trajectories reveal the distinct conformations sampled by the arginine handle in the apo *vs*. holo HCTs: Fig 2a depicts the largest cluster from the clustering analysis of the arginine handle in each simulation, while Fig 2b shows the residue’s occupancy map generated on a 3D grid and averaged over each Anton trajectory. Together, they indicate that compared with its apo state, the arginine handle is not only more internally oriented but also considerably less flexible across all five holo HCTs. Adopting primarily an internal conformation, the holo state arginine handle anchors the native substrate shikimate at the catalytic center, explaining its essential role in the acyl transfer reaction [12, 13]. We should add that although overall the arginine handle formed stable salt bridges with shikimate, our microsecond-long trajectories recorded the occasional loss of these interactions. Given this observation, we performed an extra copy of holo simulation for each HCT in order to improve sampling statistics. Among the ten holo HCT simulations listed in S2 Table, the loss of Arginine handle-shikimate interaction was observed in two trajectories: one in AtHCT and the other in CbHCT, with the salt bridges broken at *t* = 765 ns and *t* = 925 ns, respectively. In both cases, shikimate left the active site shortly afterwards, and the then free arginine handle began to swing externally, resembling its dynamics in the apo state.

**Fig 3:**
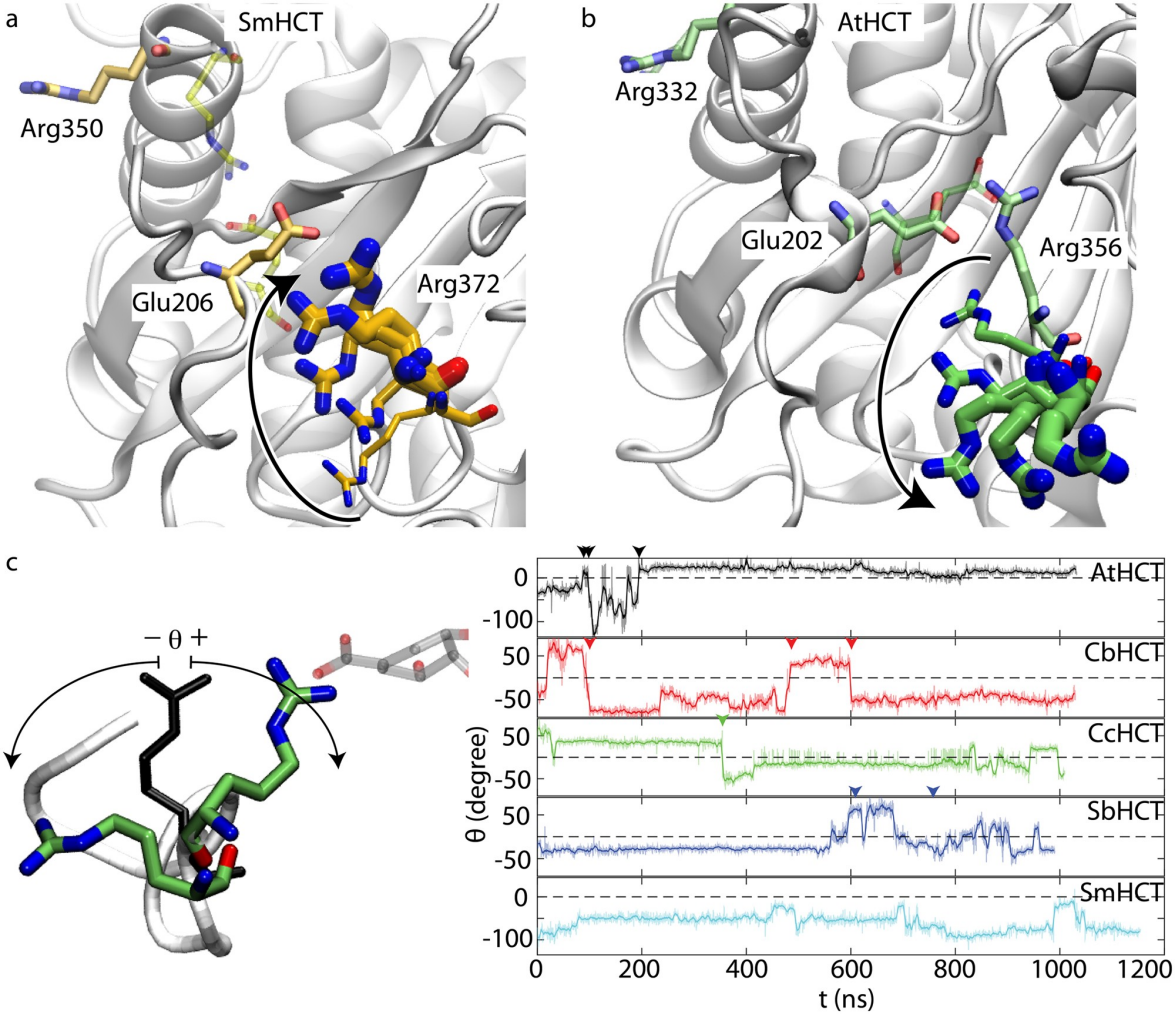
Swing motion of the arginine handle. (a-b) Simulation snapshots of the arginine handle in SmHCT (Arg372) and AtHCT (Arg356), respectively. Thicker sticks represent positions of the residue as time increases. (c) The swing angle *θ* in apo HCT simulations. For SmHCT, results from the simulation with a shikimate initially added into bulk water are shown. The apo AtHCT crystal structure, which is used as a reference to compute *θ*, is colored in black. Transition between the internal and external states of the arginine handle is marked by arrow heads (see SI for details).

As described earlier, the crystal structure of apo SmHCT contains an arginine handle more flexible than that of other HCTs. Given that this residue is indispensable for catalysis [12, 13], it must be capable of a sufficiently rapid conformational switch upon shikimate binding, *i.e*., retracting its protruded backbone and swinging its side chain, regardless of its initial position, into the internal conformation shown in Fig 2a. While this conformational switch can be enforced by SA simulations mentioned above, these simulations cannot provide any information on its timescale. Such a switch was also absent in the 1-*μ*s apo SmHCT simulation, during which the arginine handle was found to be extremely flexible, resulting in a large 1% occupancy isosurface enclosing the space visited by its side chain and a small 50% occupancy isosurface containing primarily its backbone (Fig 2b). In light of the above results, we launched a 1-*μ*s simulation of SmHCT with a free shikimate initially placed in bulk water. During this simulation, the arginine handle was found to spontaneously retrieve into the active site, a process partly mediated by a salt bridge with the highly conserved Glu206 (Fig 3a). Although shikimate did not successfully enter the active site and the arginine handle eventually swang back out, this simulation revealed the potential pathway and timescale of the residue in its switch from the apo to the holo state. Indeed, in a separate, 0.7-*μ*s SmHCT simulation with *p*-coumaroyl-CoA and shikimate manually placed into the active site (without simulated annealing), the initially outward facing arginine handle underwent a similar swing-in motion at *t* ≈ 150 ns (S3 Fig). Remarkably, a transient swing-out motion, essentially opposite to the swing-in motion described above, was recorded in apo AtHCT (Fig 3b). Although the arginine handle swung back in shortly after, its maximum root mean square deviation reached 13.2 Å, demonstrating the large scale of the transient conformational change.

The above results indicate that the arginine handle in HCT is capable of considerably more dramatic swing motions than those revealed by our previous crystal structures and short MD trajectories [10]. Indeed, the total volume of space visited by the residue ranged from 1367 Å^3^ (CcHCT) to 3415 Å^3^ (SmHCT) in the apo simulations (S3 Table). Furthermore, the swing motion of the arginine handle readily occurred on the submicrosecond timescale—as shown in Fig 3c, we characterized the internal and external states of the residue with a swing angle *θ*. In general, the transition between the two conformational states was found to take at least hundreds of nanoseconds, explaining its absence in our previous, 100-ns trajectories [10]. Although our limited number of samples precludes an accurate measure of the transition rate, averaging across all HCTs yields an estimate of ∼1.7 *μ*s^−1^. Given that the turnover rate of HCT is about 5 orders of magnitude slower, the transition of the arginine handle between its internal and external states is clearly fast enough to be mechanistically relevant for the acyl transfer reaction catalyzed by the enzyme.

### Binding dynamics of the non-native 3-hydroxyacetophenone

**Fig 4:**
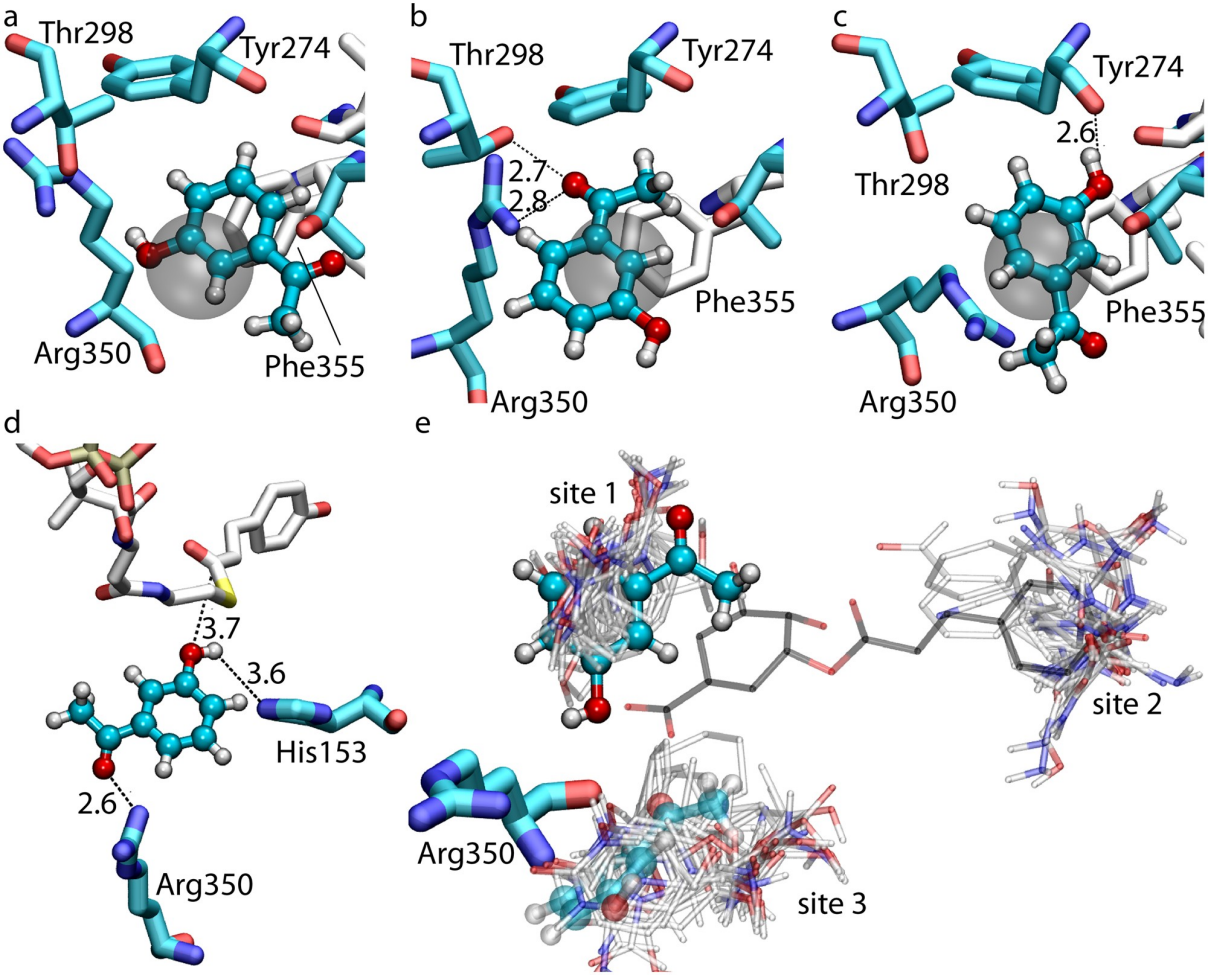
Binding dynamics of the non-native substrate 3-HAP. (a-c) The binding site of 3-HAP revealed by the CbHCT crystal structure (a) and the largest two clusters from 1-*μ*s Anton simulation (b,c). The gray surface shows a 1.7-Å-radius sphere centered at the peak of the 3-D occupancy map of 3-HAP COM. (d) Simulation snapshot at *t* = 543 ns showing the nearly productive pose of 3-HAP. (e) FTMAP results of all five HCTs. Probe molecules are shown in transparent. Conformations of 3-HAP in the crystal structure and at *t* = 737 ns of the Anton simulation are shown as solid and transparent sticks and balls, respectively. For reference, the native product *p*-coumaroylshikimate from holo AtHCT crystal structure is also shown.

HCTs are known for their considerable substrate permissiveness [10, 14]. In terms of acyl acceptor, they can process ligands with significant structural differences from the native substrate shikimate. In our previous work, we crystallized CbHCT in complex with its non-native acyl acceptor substrate, 3-HAP. As shown in Fig 4a, the ligand is coordinated by Arg350, Thr298 and Tyr274, as well as a number of non-polar residues in this non-productive pose. However, with its hydroxyl located over 8 Å away from *p*-coumaroyl-CoA and the catalytic His153, it is unclear how the acyl transfer reaction of this non-native substrate would proceed. To explore this process, we performed a 1-*μ*s simulation of CbHCT in complex with *p*-coumaroyl-CoA and 3-HAP. The crystal structure and the largest two clusters of 3-HAP from the simulation trajectory are shown in Fig 4a–4c. Together, they indicate that the ligand manifests considerable flexibility at its binding site and forms a limited number of specific interactions with the protein: apart from hydrophobic interactions with Phe355, 3-HAP may form hydrogen bonds with the side chains of Arg350 and Thr298, or, the backbone of Tyr274 after a nearly 180° rotation. This level of flexibility and the lack of more specific interactions hints that binding of 3-HAP is not particularly strong. Indeed, this non-native substrate was found to wander around in the enzyme lumen during the 1-*μ*s simulation and considerably alter both its orientation and center-of-mass (COM) position, a behavior in clear contrast to that of shikimate (S4 Fig). At *t* = 543 ns, 3-HAP wandered into a nearly productive pose, with its hydroxyl positioned within 3.7 Å and 3.6 Å of *p*-coumaroyl-CoA and His153, respectively (Fig 4d). The molecule was also stabilized by a hydrogen bond with the arginine handle, which lasted approximately 190 ns. This fleeting, nearly productive pose explains the reactivity of 3-HAP, *i.e*., despite initially bound in a non-productive pose, the non-native substrate can escape from its relatively weak binding site to explore the interior of the enzyme until it reaches its acyl transfer partner *p*-coumaroyl-CoA and catalytic residues from HCT.

Notably, the binding site of 3-HAP coincides with one of the sites (site 1) predicted by FTMAP (Fig 4e). FTMAP [15, 16] distributes 16 types of small organic probe molecules on the surface of a macromolecule to map out its binding ‘hot spots’. Regions that bind multiple probe clusters are identified as consensus sites, *i.e*., the hot spots [15]. We overlapped the consensus sites of all apo HCT crystal structures and identified three major sites across the five HCTs (with the exception of CcHCT, which did not have site 1). A common site 2 was found to overlap with the binding site of the pantothenate moiety of *p*-coumaroyl-CoA. Interestingly, a third site (site 3) was found near the arginine handle and was briefly visited by 3-HAP during the 1-*μ*s Anton simulation. In addition to the strictly conserved Arg350, residues surrounding site 3 include Val30, Pro32, and Asn294, all of which are highly conserved across HCT orthologs [10]. It is worth mentioning that the formation of this site depends on the conformation of the arginine handle: in the crystal structures and Anton trajectories of holo HCTs, site 3 disappears due to the more internally oriented arginine handle.

### Off-center binding and reaction rate

Given the binding dynamics described above, an intriguing question arises: how big an effect on the reaction rate, if any, can we expect from the presence of an off-center binding site? Qualitatively, the presence of a binding site, even an off-center one, should increase the probability of 3-HAP visiting the enzyme active site, thereby, facilitating its reaction; however, a long residence time at this site risks trapping the molecule at a location far away from the catalytic residues, thereby, interfering with its reaction. Assuming that substrates do not interact with each other, a previous study on channel transport suggests that the former effect should be dominating [17]. Multiple studies on protein-ligand binding [18–21], many of which focused on the electrostatic interactions between charged ligands and an enzyme, also point at a facilitating role of an attractive potential. Nonetheless, as these studies are not tailored for promiscuous enzymes, they do not provide a quantitative answer to the question raised above.

**Fig 5:**
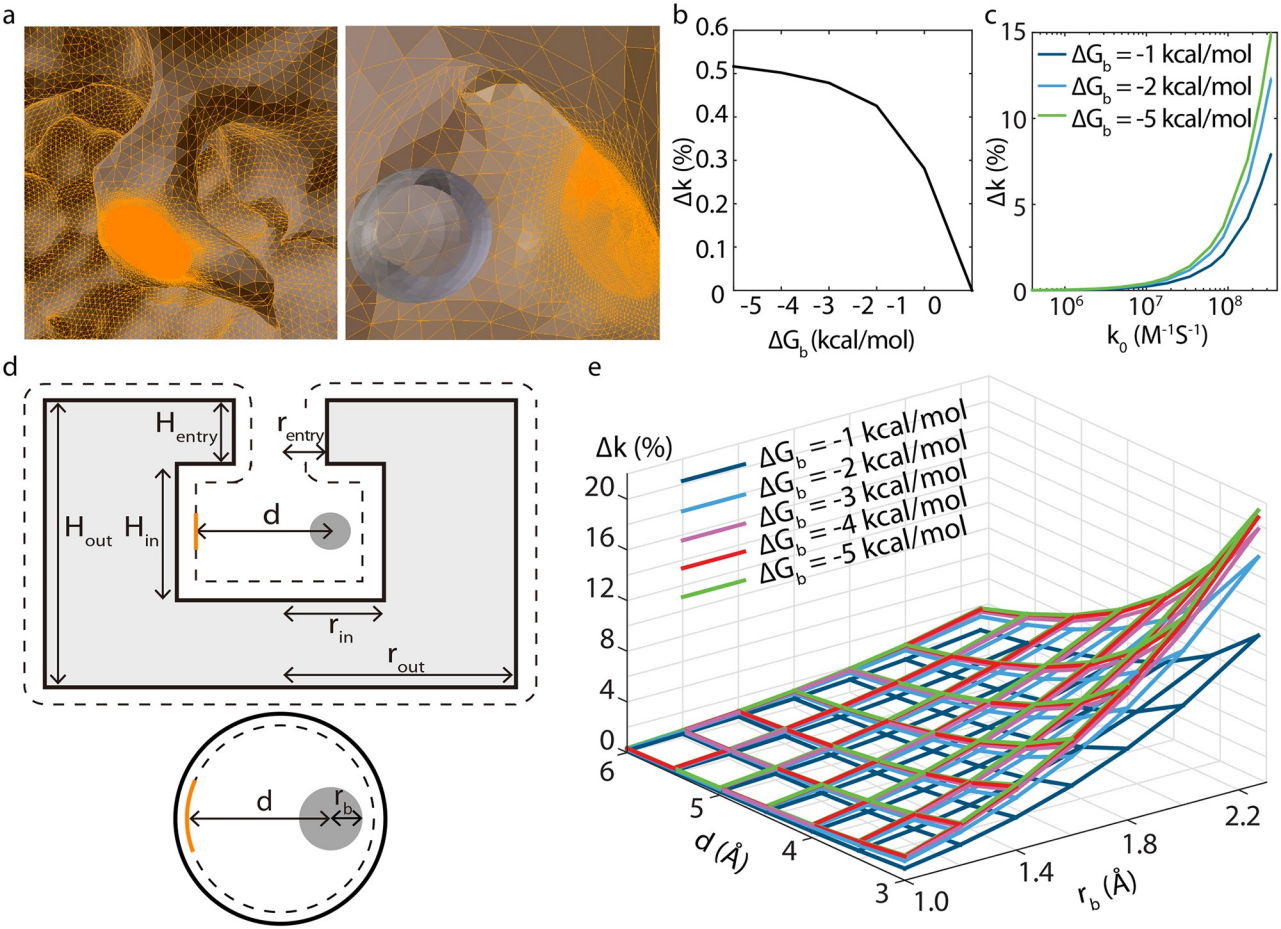
Reaction rate increase (Δ*k*) brought by off-center binding in CbHCT (a-b) and a cylindrical enzyme model (c-e). (a) Side (left) and top (right) view of the CbHCT active site. The molecular mesh of CbHCT is colored in orange, with the surface of the catalytic center having the highest mesh density. The off-center binding site is represented by a gray sphere. (b) Δ*k* as a function of the 3-HAP binding site affinity. (c) Δ*k* as a function of the the base reaction rate *k*_0_ in the cylindrical enzyme model, with *H*_*in*_ = 9 Å, *r*_*in*_ = 6 Å, *r*_*b*_ = 2 Å, and *d* = 3 Å. The reactivity parameter *α* is scanned so that *k*_0_ varies from around 10^6^ M^−1^S^−1^ to 10^8^ M^−1^S^−1^. Δ*k* at a given *α* is plotted against the corresponding *k*_0_. (d) Schematics of the cylindrical enzyme model. Dashed lines outline its molecular surface. (e) Δ*k* as a function of the radius *r*_*b*_, the affinity Δ*G*_*b*_ of the off-center site, and its distance to the catalytic center *d*. Calculation performed with *α* = ∞, *H*_*in*_ = 9 Å and *r*_*in*_ = 6 Å.

Here, we quantify the influence of an off-center binding site on the reaction rate of a neutral, non-native substrate in both HCT and a generic, cylinder-shaped enzyme model. Using the equilibrated structure of CbHCT in complex with *p*-coumaroyl-CoA, we first created the molecular mesh of the enzyme (Fig 5a) and then solved the steady-state Smoluchowski equation (SSSE) characterizing the diffusion of the substrate. The reaction rate (*k*) was determined by integrating the flux of the substrate over the surface of the catalytic center, which had a radiation boundary condition [22–28]. With an intrinsic reactivity parameter *α* representing how ‘good’ the enzyme was, all other chemical details were hidden from our calculation. The relative change in the reaction rate (Δ*k*) was obtained by solving the SSSE first without and then with the 3-HAP binding site, which was modeled as a 1.7-Å-radius sphere (Fig 4) based on analysis of the Anton trajectory (see SI for details). Its affinity for 3-HAP was varied from -5 to -1 kcal/mol, *i.e*., from relatively strong to extremely weak, to examine how binding strength at this site affected Δ*k*.

Our calculations indicate that the presence of the off-center binding site always increases the reaction rate of 3-HAP. In essence, this enhancement is analogous to the channel transport case reported by Bauer and Nadler [17], *i.e*., an increased probability of the substrate visiting the catalytic center, brought by the presence of an off-center site, outweighs the prolonged first passage time caused by the ‘trapping’ effect of this site. The magnitude of such rate enhancement, as further demonstrated below in the cylindrical enzyme model, depends on the size and location of the off-center site, the geometry of the enzyme lumen as well as the reactivity of the enzyme. For 3-HAP, Δ*k* could only reach 0.5% even if we assumed that its reaction was diffusion-limited (*α* = ∞). This result can be attributed to a rather large separation between the 3-HAP binding site and the catalytic center as well as the tunnel-shaped HCT lumen. In reality, as the acyl transfer reaction between 3-HAP and *p*-coumaroyl-CoA is far from the diffusion limit [10], Δ*k* brought by the off-center binding site should be negligible.

Despite the small Δ*k* observed above, it is of interest to systematically examine various physico-chemical properties controlling the impact of an off-center binding site. Thus, we went on to study a generic, cylindrical enzyme model, the volume of which was chosen to match a typical enzyme cavity volume of 1000 Å^3^ [29]. The height (*H*_*in*_) of the cylinder and the radius of its base (*r*_*in*_) were varied, while its volume was kept approximately constant. For a given pair of *H*_*in*_ and *r*_*in*_, we scanned the affinity (Δ*G*_*b*_) and size (*r*_*b*_) of the off-center site as well as its distance to the catalytic center (*d*). Setting Δ*G*_*b*_ to zero yields the base reaction rate of the enzyme (*k*_0_), which is on the order of 10^8^ M^−1^s^−1^ when *α* is infinite, *i.e*., the reaction is diffusion-limited [30].

As shown in Fig 5c, Δ*k* depends strongly on the reactivity of the enzyme. Only when the reaction is limited or heavily influenced by diffusion (*k*_0_ > 10^7^ M^−1^s^−1^), can Δ*k* become significant. In these cases, the effect of the off-center site closely depends on its distance to the catalytic center—the smaller *d* is, the larger Δ*k* becomes (Fig 5e). Additionally, the geometry of the enzyme lumen matters: as the cylindrical model elongates, Δ*k* decreases (S5a Fig). In the limiting case, our cylinder should approach the 1D model of Berezhkovskii et al. [31]. The decrease in Δ*k* observed here is analogous to a decreasing contribution from the internal domain of the 1D model, *i.e*., as the enzyme lumen narrows, finding its entrance from the outside becomes too slow and the presence of a binding site within the active site no longer significantly accelerates the rate at which the substrate reaches the catalytic center. For this reason, off-center binding is less likely to be important in a tunnel-shaped enzyme compared with a basin-shaped one with a wide opening to its active site.

**Fig 6:**
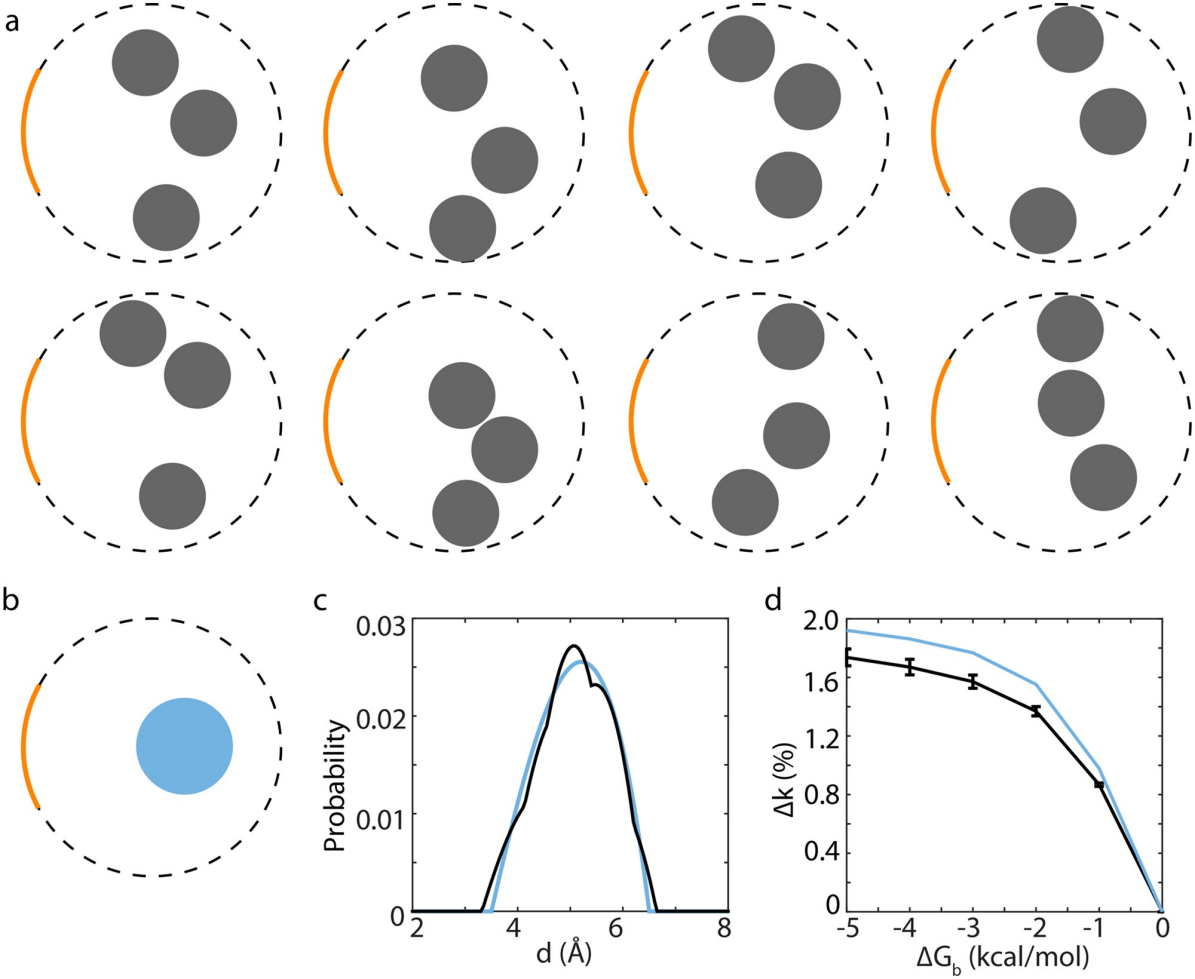
Effect of splitting an off-center binding site. (a-b) Eight randomly selected configurations of three off-center sites (a) with a total volume same as the single off-center site shown in (b). The absorbing surface of the catalytic center is colored in orange. The cylindrical enzyme model has *H*_*in*_ = 9 Å and *r*_*in*_ = 6 Å. (c) Distributions of the distance (*d*) from all points within the three sites (black) or the single site (blue) to the catalytic center. Locations of the three sites have been chosen so that the two *d* distribution profiles match as closely as possible (see SI for details). (d) Δ*k* produced by the three sites (black) and the single off-center site (blue). The calculations were performed with *α* = ∞. Results from all eight configurations shown in (a) are averaged, with error bars depicting their standard deviations.

Most interestingly, with other metrics held constant, the size of the off-center binding site, rather than its strength, dominated Δ*k*. For instance, in the 3D plot shown in Fig 5e, strengthening the site’s affinity from -2 to -4 kcal/mol with *α* = ∞, *d* = 3 Å and *r*_*b*_ = 1.8 Å only brought Δ*k* from 9% to 10%. In contrast, doubling the volume of the binding site yielded Δ*k* = 18% even with Δ*G*_*b*_ staying at -2 kcal/mol. Furthermore, splitting the binding site into multiple sites while keeping the same total volume does not affect Δ*k* significantly (Fig 6). In general, we found that with other metrics held constant, Δ*k* scaled linearly with the volume of an off-center site, whereas its relation with Δ*G*_*b*_ was approximately an exponential one (S5 Fig). Thus, rather than enhancing the affinity of an off-center binding site, developing larger and/or additional sites, even ones with only weak affinity, appears to be a more effective strategy for a promiscuous enzyme to speed up the reaction of its non-native substrates.

## Discussion

Combining X-ray crystallography and microsecond-long MD simulations, we investigated plant HCTs’ substrate permissiveness by examining their active-site dynamics in the apo state and upon the binding of their native as well as non-native acyl acceptor substrates. A prevalent swing motion of the arginine handle was observed across all HCTs, the timescale of which was found to be on the order of sub-microsecond, *i.e*., sufficiently fast to facilitate the recognition of HCT substrates *in vivo*. Apart from stabilizing the native substrate shikimate in its reactive pose, the arginine handle formed a transient hydrogen bond with the non-native, neutral 3-HAP as the latter adopted a nearly productive pose. The conformation of this residue also dictated the formation of an additional binding hot spot (site 3) briefly visited by 3-HAP, suggesting that it may enable HCTs to provide multiple binding sites for their non-native substrates. Utilizing a flexible arginine to recognize different substrates is a known strategy to sow the seeds for promiscuity. One prominent example is the amine transaminase, where an arginine can flip away to create a cavity to accommodate even substrates lacking a carboxylate group [32]. Given the remarkable flexibility of the arginine handle in HCTs, it appears that a similar strategy has been adopted by these enzymes—with the residue sweeping across the active site on a sub-microsecond timescale, HCTs can readily accommodate its native and various non-native substrates of different sizes, with or without a carboxylate group.

Among the five HCTs studied here, clear differences exist in the extent of the arginine handle’s flexibility. For instance, SmHCT presents an externally protruded backbone of Arg372 in the apo state, a distinguishing feature compared to the remaining HCTs. Considering the total volume of space visited by the residue during Anton simulations (S3 Table), we found that CcHCT and SmHCT appeared to be at the two ends of the flexibility spectrum, with the arginine handle being only moderately flexible in the former and exhibiting large-scale swing motion in the latter. Such differences are not entirely unexpected: While HCTs share a common feature of substrate permissiveness, each has its unique promiscuity profile. For instance, the activity of AtHCT towards the non-native acyl acceptor naringenin is impaired relative to that of CbHCT and SmHCT (S6 Fig). While the origin of their differential promiscuity profiles remains to be fully understood, given the central role of the arginine handle in substrate recognition, its different conformational flexibility among various HCTs likely plays a part.

The non-native substrate 3-HAP binds HCT at a site approximately 8 Å away from the catalytic center. This off-center site was identified as a hot spot (site 1) by FTMAP, suggesting that it could also serve as a binding site for other non-native acyl acceptor substrates. Our simulations revealed that this site had a relatively weak affinity for 3-HAP, allowing the molecule to leave and explore the interior of the enzyme, eventually achieving a nearly productive pose during a 1-*μ*s simulation. This pose is only stabilized by a hydrogen bond between the carbonyl oxygen of 3-HAP and the arginine handle, which lasted approximately 190 ns, *i.e*., considerably weaker than the salt bridge formed between the latter residue and the native substrate shikimate. This difference reflects the competitiveness of the native substrate over the non-native one, affirming the findings of our previous study [10].

The binding dynamics of 3-HAP raises the intriguing question regarding the role of an off-center binding site. Intuitively, one may expect (correctly) that binding at an off-center site serve to increase the presence of the substrate within the enzyme lumen, which should lead to its enhanced probability of visiting the catalytic center. However, it is nontrivial to determine whether such an enhancement will always speed up the corresponding reaction, since an off-center site also risks trapping the substrate at a location away from the catalytic center, resulting in a prolonged first passage time [17]. Furthermore, exactly how big an effect (if any) can be expected from an off-center site cannot be deduced from qualitative arguments. By solving the steady-state Smoluchowski equation first for HCT and then for a generic enzyme model, we show that the reaction rate of a given substrate is always enhanced by the presence of an off-center binding site, *i.e*., Δ*k* > 0; however, its large distance to the catalytic center as well as the relatively narrow entrance of the HCT lumen dictates that Δ*k* brought by the 3-HAP binding site is capped at ∼0.5%. The actual Δ*k* is likely much smaller given that the reaction of 3-HAP is far from the diffusion limit. In general, only when a reaction is limited or heavily influenced by diffusion (*k*_0_ > 10^7^ M^−1^s^−1^), can Δ*k* become significant. Otherwise, with diffusion faster than catalysis, on average a substrate will always be around when the reaction occurs, with or without the presence of an off-center binding site.

For reactions heavily influenced by diffusion, a significant rate enhancement can be achieved by off-center binding. Notably, even a relatively weak binding site (-2 to -3 kcal/mol) can already produce a non-negligible Δ*k*. Indeed, our calculations indicate that Δ*k* depends more strongly on the size of the site rather than its strength. Furthermore, splitting the binding site while keeping the same total volume does not significantly affect the resulting Δ*k*. These data suggest that having multiple, weak binding sites can be highly desirable for a non-native substrate. From an evolutionary perspective, it may in fact be less challenging to develop these sites than a single, strong binding site, since the latter tends to require highly specific interactions. Overall, while stabilizing the transition states remains the key to enabling non-native reactions, providing off-center binding sites may constitute a low-barrier mechanism to facilitate substrate permissiveness by certain enzymes. If favorable, such activities can be refined through rounds of mutation, duplication, and selection to yield enzymes with novel functions and unique molecules in the network of specialized metabolism.

## Materials and methods

The Protein Data Bank accession number of SmHCT crystal structure is 6DD2. X-ray diffraction intensities were indexed and integrated with iMosflm [33] and scaled with Scala under CCP4 [34, 35]. The phase was determined with molecular replacement using Phaser under Phenix [36]. Coot was used for manual map inspection and model rebuilding [37]. Anton simulations performed in this work are listed in S2 Table. Prior to the Anton runs, all systems were subjected to a 20 ns equilibration performed with NAMD 2.10 [38]. The missing loops in CcHCT, SbHCT and SmHCT were modeled using Modeller [39]. CHARMM36 [40] and CHARMM General force field [41] were employed for all simulations. Parameters of the CoA moiety from ref [42] were used, while parameters of shikimate and the *p*-coumaroyl moiety were obtained from the CGenFF program [43, 44] and further validated using Gaussian [45] and the Force Field Toolkit [46] plugin of VMD [47]. Clustering analysis was performed with GROMACS [48] and structural alignment was done with the Multiseq [49] plugin of VMD. SSSE calculations were performed using Mathematica (version 11.2) [50], with the molecular meshes of CbHCT and the cylindrical enzyme model created using PyMOL [51] and Blender [52]. See SI for more details.

## Supporting information

S1 Text Crystallization, MD simulation, SSSE calculation as well as other calculation and analysis protocols are provided. (PDF)

S1 Data MD simulation input files, including the topology and structure of each system, the force field parameters of shikimate, 3-HAP and *p*-coumaroyl-CoA as well as the Anton configuration files are provided in the supporting information. SSSE mesh files and Mathematica scripts for both CbHCT and the cylindrical enzyme model are also provided.
(ZIP)

S1 Fig Reaction mechanism (a) and native (b) as well as an example of non-native reaction (c) catalyzed by HCT. (a,b) The *p*-coumaroyl group is transferred from *p*-coumaroyl-CoA to the native substrate shikimate, resulting in the product *p*-coumaroylshikimate. Residues are numbered according to SmHCT. (c) The acyl transfer reaction occurs between *p*-coumaroyl-CoA and 3-hydroxyacetophenone.
(PDF)

S2 Fig Anton simulation system of holo AtHCT. The water box is shown as a transparent surface in (a) and the positions of *p*-coumaroyl-CoA (stick representation) and shikimate (vdW representation) are highlighted in (b).
(PDF)

S3 Fig Interactions between the arginine handle and shikimate in a 0.7-*μ*s SmHCT simulation. Shikimate was placed within the SmHCT active site manually at the beginning of the simulation. (a) Snapshot of the system at *t* = 147 ns. The position of Arg372 at *t* = 0 ns is shown in thin sticks for reference. (b) Distance between the central carbon of the guanidinium group in Arg372 and the carbon of the carboxyl group in shikimate.
(PDF)

S4 Fig Orientation and center-of-mass (COM) position of shikimate (a) and 3-hydroxyacetophenone (b) in Anton simulations of holo CbHCT. Both the orientation and COM displacement are with respect to the CbHCT crystal structure. Calculations details as described in our previous work [10].
(PDF)

S5 Fig Impact of geometry, size and strength of the off-center binding site on Δ*k*. (a) Δ*k* decreases as the cylindrical enzyme elongates. The volume of the cylinder was kept approximately constant. The calculations were performed with *r*_*b*_ = 2 Å, *d* = 3 Å and *α* = ∞. (b-c) With other metrics held constant, Δ*k* scales linearly with the volume (*V*_*b*_) of the off-center site (b), and has an exponential dependence on the binding affinity (Δ*G*_*b*_) of the site (c). The dots represent calculation results obtained with *H*_*in*_ = 9 Å, *r*_*in*_ = 6 Å, *d* = 3 Å and *α* = ∞. The curves represent fitting results obtained with a linear (b) and an exponential (c) function, respectively.
(PDF)

S6 Fig Promiscuous activity of AtHCT, CbHCT and SmHCT against 1 mM of 2,3-dihydroxybenzoic acid, naringenin, *p*-coumaric acid, and 3-hydroxyacetophenone. Reactions were incubated overnight, and product formation measured via liquid chromatography-mass spectrometry. Reaction conditions as previously described [10].
(PDF)

S1 Table Selected residue IDs in HCT orthologs. (PDF)

S2 Table Anton simulations performed in this work. (PDF)

S3 Table Total volume of space visited by the arginine handle in apo-HCT simulations. (PDF)

S4 Table Refinement statistics of the SmHCT crystal structure. (PDF)
