# Supplementary material for: Structural and dynamic basis of substrate permissiveness in hydroxycinnamoyltransferase (HCT)
Source: PLoS Comput Biol. 2018 Oct 26;14(10):e1006511. doi: 10.1371/journal.pcbi.1006511 (PMC6203249; doi:10.1371/journal.pcbi.1006511)
Supplement: S1 Text — (PDF) [file pcbi.1006511.s001.pdf]

Supporting information

Crystallization protocols

SmHCT crystals were grown by hanging drop vapor diffusion at 4 ^{o}C by mixing 2 μL 10.2 mg/mL protein with 1 μL of a reservoir solution containing 0.1 M HEPES:NaOH, 2 M ammonium sulfate, pH 6.5. Crystals were transferred to a cryoprotection solution of 17% glycerol in reservoir solution. Single crystals were mounted in a cryoloop and flash frozen in liquid nitrogen. X-ray diffraction data for SmHCT was collected at beamline 24-ID-E of the Advanced Photon Source at Argonne National Laboratory on a CCD-based ADSC Quantum 315 detector. Structural refinement and crystallographic calculations were performed using the Phenix program (1). Refinement statistics of the SmHCT structure are provided in Table S4.

MD simulation protocols

All simulation systems were constructed from the crystal structures of apo and holo HCTs (2–4). For HCTs lacking a holo structure, namely, CbHCT, CcHCT and SmHCT, their corresponding apo structures were utilized and shikimate and *p*-coumaroyl-CoA were added according to positions suggested by the *p*-coumaroyl-CoA-bound and *p*-coumaroylshikimate-bound AtHCT under alignment (5). To fully relax residues around the newly added substrates, a series of simulated annealing simulations (SA) were performed before the holo state Anton simulations of the above systems were launched. The SA protocols used here were identical to those adopted in our previous work (2). Specifically, the temperature was increased from 300K to 500K at a speed of 10K/10ps; after a 5-ns NPT equilibration, the temperature was reduced to 300K at a speed of 10K/200ps, which was followed by a 1000-step minimization. During the SA simulations, substrate heavy atoms and protein backbones were restrained, while atoms on loops L1-L3 (Fig 1 a) were allowed to move freely. For SmHCT, the missing loop (resid 216-239) reconstructed with Modeller (6) was also allowed to move freely. The SA simulations were repeated fives times for each system. The centroid structure obtained from a clustering analysis over the five SA replicas was taken as the initial structure for subsequent Anton simulations. As a control, the same SA protocol was applied on apo AtHCT, which produced a structure highly similar to the holo AtHCT crystal structure with a RMSD of 0.8 Å for the arginine handle. Except for the above systems that went through simulated annealing, all other systems were subjected to a 5-ns restrained equilibration, with protein backbones (excluding missing residues reconstructed with Modeller (6)) and substrate carbon atoms restrained with a force constant of 3.0 kcal/mol/Å^2. Finally, all systems were subjected to a 20 ns equilibration prior to Anton runs. Anton simulations were performed under the isotropic NPT conditions (1 atm and 300 K) using the Multigrator integrator and a timestep of 2 fs. The bonded forces were updated every step, and the nonbonded near and far forces were updated every 1 and 3 steps, respectively. Trajectories were saved every 240 ps. Viparr 4.4.12 was used in system preparation and all calculations were performed using anton software version 2.13.0. The complete list of Anton simulations performed in this work is provided in Table S2. Apart from these Anton runs, an additional simulation was performed on SmHCT in complex with shikimate and *p*-coumaroyl-CoA. The apo SmHCT crystal structure was utilized, with the substrates introduced into the active site by the same alignment protocol described above. No simulated annealing was performed for this system and after a 5000-step restrained minimization it was simulated for 700 ns

with NAMD 2.10 (7).

SSSE calculation protocols

The steady-state Smoluchowski equation takes the form

$$\nabla \cdot De^{-\beta U(\mathbf{r})}\nabla e^{\beta U(\mathbf{r})}\rho(\mathbf{r}) = 0 \tag{1}$$

where D is the diffusion constant of the moving ligand, $\beta = 1/k_B T$ with k_B representing the Boltzmann constant and T the temperature. $U(\mathbf{r})$ is the ligand's potential of mean force and $\rho(\mathbf{r})$ is its concentration at position $\mathbf{r}$. In this work, we assumed that $U(\mathbf{r})$ was zero except at the specified binding site. More specifically, $U(\mathbf{r})$ adopted a form similar to a step function (8, 9)

$$U(\mathbf{r}) = -\frac{\Delta G_b}{2}(tanh\frac{\|\mathbf{r}-\mathbf{r}_b\| - r_b}{L} - 1) \tag{2}$$

with ΔG_b standing for the site's binding affinity, $\mathbf{r}_b$ the coordinate of the binding site center and r_b the radius of the spherical binding site. The constant L was chosen according to $L/r_b = 0.05$, which yielded a smooth transition of $U(\mathbf{r})$ from ΔG_b to zero across the boundary of the binding site.

A radiation boundary condition was adopted at the surface of the catalytic center (Γ_a):

$$\mathbf{n} \cdot \mathbf{J}(\mathbf{r}) = \alpha\rho(\mathbf{r}), \mathbf{r} \in \Gamma_a \tag{3}$$

with $\mathbf{J}(\mathbf{r})$ standing for the flux of the ligand, $\mathbf{n}$ the surface normal along Γ_a, and α the intrinsic reactivity of the enzyme measuring how quickly a ligand is consumed upon reaching the surface of catalytic center. Other boundary conditions were identical to those adopted in previous studies(10–12). We should add that the enzyme was kept rigid throughout our SSSE calculations. Protein flexibility, which has been treated in recent studies (9, 13), may be explored in our future work.

The diffusion-influenced reaction rate k was obtained as the area integral of the flux crossing the surface of the catalytic center

$$k = \frac{\int_{\Gamma_a} \mathbf{n} \cdot \mathbf{J}(\mathbf{r}) ds}{\rho_{bulk}} \tag{4}$$

with ds standing for the surface area element along Γ_a and ρ_{bulk} the bulk concentration of the ligand.

All SSSE calculations were performed with the finite element package of Mathematica (version 11.2) (14). Molecular meshes of CbHCT and the cylindrical enzyme model were generated using Pymol (15) and Blender (16). For CbHCT, the molecular mesh was created by rolling a sphere of radius $\sigma = R_{vdW} + R_{lig}$ along the protein, with R_{vdW} standing for the vdW radius of an atom according to the CHARMM force field (17) and R_{lig} taken to be the radius of a carbon atom (1.7 Å), which corresponded to the 'thinnest' part of the 3-HAP molecule. The center of the catalytic surface was defined as the mid point between N_ε of the catalytic His153 and the carbon atom from the carbonyl group in the *p*-coumaroyl moiety of *p*-coumaroyl-CoA. Assuming that the hydroxyl oxygen of 3-HAP resided at this point during the acyl transfer reaction, the COM of 3-HAP would be 2.8 Å away. Therefore, the portion of the CbHCT molecular mesh within a 2.8-Å sphere centered at this point was taken to be the catalytic surface. The 3-HAP binding site was modeled

as a sphere centered at the peak of the 3D occupancy map of the molecule's COM during the 1-μs Anton simulation. The radius of this sphere ($r_b = 1.7$ Å) was chosen so that its volume matched the volume of the convex hull formed by all COM positions of 3-HAP from the top two clusters of the Anton trajectory (Fig 4 b-c).

For the cylindrical enzyme model, R_{lig} was set to 2 Å and the catalytic surface was defined as the portion of the enzyme molecular mesh within a 2-Å sphere centered at the midpoint of its cylindrical wall (Fig 5 d). The off-center binding site resided on the same horizontal plane as the catalytic center, while their distance (d) and the radius of the off-center site (r_b) were varied. The volume of the enzyme lumen was kept approximately constant at 1000 Å^3. A small 'neck' region ($H_{entry} = 3$ Å, $r_{entry} = 5$ Å) was introduced to mimic the entrance of an enzyme lumen (Fig 5 d). The following set of parameters were utilized in the calculation shown in S5 a: H_{in} =8, 9, 10, 11 Å and r_{in} =6.4, 6, 5.7, 5.4 Å, respectively. During the calculation of Δk upon splitting an off-center site shown in Fig 6, in order to keep other metrics as similar as possible between the single-site and multiple-site cases, the distance distribution profiles from all points within the sites were matched. This was achieved by maintaining the same total volume of the sites while minimizing the RMSD between the two distance distribution profiles shown in Fig 6 c. For a 1.5-Å single site placed at $d = 5$ Å (Fig 6 b), the best match was obtained when three 1.04-Å sites were placed at $d = 4.36$, 5.17, and 5.6 Å, respectively. A Monte Carlo (MC) calculation was then performed to randomly place these three sites within the enzyme lumen, subject to the above distance constraint. Eight configurations emerged from the 10,000-round MC calculations, which are shown in Fig 6 a.

Other calculations and analysis

Clustering analysis of the arginine handle and 3-HAP was both performed using gmx cluster (version 2016.2) from the GROMACS package (18). The gromos clustering method was used with a cutoff value of 2.0 Å. The swing angle θ was measured by first defining a vector pointing from the Cα atom of the arginine handle to the central carbon of its guanidinium group. θ was then determined as the angle between this vector and its reference in the apo AtHCT crystal structure. To characterize the arginine handle's transition between its internal and external states, a histogram of θ was obtained and the number of transitions between the positive and negative peak values of θ was recorded. The number of transition events were divided by the simulation time and averaged across all five HCTs, yielding an estimated average transition rate. FTMAP analysis (19, 20) was performed via the web server http://ftmap.bu.edu/login.php with default settings.

References

1. Adams PD, Afonine PV, Bunkóczi G, Chen VB, Davis IW, Echols N, et al. PHENIX: a comprehensive Python-based system for macromolecular structure solution. Acta Crystallogr D Biol Crystallogr. 2010;66(2):213–221.

2. Levsh O, Chiang YC, Tung CF, Noel JP, Wang Y, Weng JK. Dynamic Conformational States Dictate Selectivity toward the Native Substrate in a Substrate-Permissive Acyltransferase. Biochemistry. 2016;55(45):6314–6326.

3. Lallemand LA, Zubieta C, Lee SG, Wang Y, Acajjaoui S, Timmins J, et al. A structural basis for the biosynthesis of the major chlorogenic acids found in coffee. Plant Physiol. 2012;160(1):249–260.

4. Walker AM, Hayes RP, Youn B, Vermerris W, Sattler SE, Kang C. Elucidation of the structure and reaction mechanism of sorghum hydroxycinnamoyltransferase and its structural relationship to other coenzyme A-dependent transferases and synthases. Plant Physiol. 2013;162(2):640–651.

5. Roberts E, Eargle J, Wright D, Luthey-Schulten Z. MultiSeq: Unifying sequence and structure data for evolutionary analysis. BMC Bioinform. 2006;7:382.

6. Fiser A, Do RK, Sali A. Modeling of loops in protein structures. Prot Sci. 2000;9:1753–1773.

7. Phillips JC, Braun R, Wang W, Gumbart J, Tajkhorshid E, Villa E, et al. Scalable Molecular Dynamics with NAMD. J Comput Chem. 2005;26:1781–1802.

8. Cai L, Zhou HX. Theory and simulation on the kinetics of protein–ligand binding coupled to conformational change. J Chem Phys. 2011;134(10):03B607.

9. Greives N, Zhou HX. BDflex: A method for efficient treatment of molecular flexibility in calculating protein-ligand binding rate constants from Brownian dynamics simulations. J Chem Phys. 2012;137(13):135105.

10. Kekenes-Huskey P, Gillette A, McCammon J. Predicting the influence of long-range molecular interactions on macroscopic-scale diffusion by homogenization of the Smoluchowski equation. J Chem Phys. 2014;140(17):174106.

11. Song Y, Zhang Y, Shen T, Bajaj CL, McCammon JA, Baker NA. Finite element solution of the steady-state Smoluchowski equation for rate constant calculations. Biophys J. 2004;86(4):2017–2029.

12. Song Y, Zhang Y, Bajaj CL, Baker NA. Continuum diffusion reaction rate calculations of wild-type and mutant mouse acetylcholinesterase: adaptive finite element analysis. Biophys J. 2004;87(3):1558–1566.

13. Kekenes-Huskey P, Gillette A, Hake J, McCammon J. Finite-element estimation of protein–ligand association rates with post-encounter effects: applications to calcium binding in troponin C and SERCA. Comput Sci Discov. 2012;5(1):014015.

14. Inc WR. Mathematica, Version 11.2; 2017.

15. Schrödinger, LLC. The PyMOL Molecular Graphics System, Version 2.0.6; 2015.

16. Blender Online Community. Blender - a 3D modelling and rendering package; 2017.

17. Best RB, Zhu X, Shim J, Lopes PEM, Mittal J, Feig M, et al. Optimization of the Additive CHARMM All-Atom Protein Force Field Targeting Improved Sampling of the Backbone ϕ, ψ and Side-Chain χ_1 and χ_2 Dihedral Angles. J Chem Theory Comput. 2012;8(9):3257–3273. doi:10.1021/ct300400x.

18. Abraham MJ, Murtola T, Schulz R, Pll S, Smith JC, Hess B, et al. GROMACS: High performance molecular simulations through multi-level parallelism from laptops to supercomputers. SoftwareX. 2015;12:19 – 25.

19. Brenke R, Kozakov D, Chuang GY, Beglov D, Hall D, Landon MR, et al. Fragment-based identification of druggable 'hot spots' of proteins using Fourier domain correlation techniques. Bioinformatics. 2009;25(5):621–627.

20. Kozakov D, Grove LE, Hall DR, Bohnuud T, Mottarella SE, Luo L, et al. The FTMap family of web servers for determining and characterizing ligand-binding hot spots of proteins. Nat Protoc. 2015;10:733–755.
