# Supplementary material for: Structural and dynamic basis of substrate permissiveness in hydroxycinnamoyltransferase (HCT)
Source: PLoS Comput Biol. 2018 Oct 26;14(10):e1006511. doi: 10.1371/journal.pcbi.1006511 (PMC6203249; doi:10.1371/journal.pcbi.1006511)
Supplement: S1 Data — SSSE mesh files and Mathematica scripts for both CbHCT and the cylindrical enzyme model are also provided. (ZIP) [file pcbi.1006511.s002.zip › SI_data/02_SSSE_Calculation_Files/02_Model_Enzyme/main_model.pdf]

```
(*setting*)
SetDirectory[NotebookDirectory[]];
Needs["NDSolve`FEM`"];

kcalpermoltokBT=1/0.593;
β=1;(*1/kBT*)
rReactionCenter=2;(*radius of the active site*)
rOuterBoundary=200;(*radius of outer boundary*)
α=1000000000000000000000000;(* constant for reactive robin condition/intrinsic bimolecular rat
reactionCenterCoord= {-4,0,2.5};(*location of center of active site*)
voidCoord={0,0,-1};
```

```
(*MESH GENERATION*)
bmouter=ToBoundaryMesh[Sphere[{0,0,0},rOuterBoundary],AccuracyGoal→2];
SetDirectory[NotebookDirectory[]];
meshprotein=Import["mesh/H9_r6.stl"];
bmeshprotein=ToBoundaryMesh[meshprotein];


bmeshfinal=ToBoundaryMesh["Coordinates"→Join[bmeshprotein["Coordinates"],bmouter["Coordinates
"BoundaryElements"→Join[bmeshprotein["BoundaryElements"],
MapThread[#1[#2]&,{Head/@bmouter["BoundaryElements"],Length[bmeshprotein["Coordinates"]]+Elem
"RegionHoles"→{voidCoord}];

f=Function[{vertices,area},
 Block[{x,y,z},{x,y,z}=Mean[vertices];
If[x^2+y^2≤10^2 && -1≤z≤14,area>0.005,area>0.005+1000*Tanh[(x^2+y^2+(z-10)^2)/10.0^2]]] (* r
];

(mesh=ToElementMesh[bmeshfinal,"BoundaryMeshGenerator"→{"Continuation"},
                                                          "RegionHoles"→{voidCoord}
,MeshRefinementFunction→f
])

activesitecoorlist=Select[bmeshfinal["Coordinates"],Norm[#-reactionCenterCoord]≤rReactionCent

coordinates=bmeshfinal["Coordinates"];
incidents=Flatten[ElementIncidents[bmeshfinal["BoundaryElements"]],1];
normals=Flatten[bmeshfinal["BoundaryNormals"],1];
coordinatePairs=GetElementCoordinates[coordinates,incidents];
```

```
ElementMesh[{{-200., 200.}, {-200., 200.}, {-200., 200.}},
 {TetrahedronElement[<477295>]}]
```

```
(*Solving PDE*)
solveSMOL[{bindingSiteStrength_,κ_,rBSpotential_,Bcenter_,finalmesh_}]:=Module[{mesh,U,c,op2,
ActiveSiteArea,ActiveSiteCoor,ActiveSiteNormal,count},

mesh=finalmesh;
deltaGB=bindingSiteStrength*kcalpermoltokBT;
potshape=0.05; (* adjust the shape of binding site potential: L/rb = 0.05*)

U[x_,y_,z_]= - deltaGB/2*( Tanh[(Norm[{x,y,z}-Bcenter]-rBSpotential)/(potshape*rBSpotential)

c[x_,y_,z_]={{Exp[-β*U[x,y,z]],0,0},{0,Exp[-β*U[x,y,z]],0},{0,0,Exp[-β*U[x,y,z]]}};
op2=Inactivate[Div[-c[x,y,z].Grad[u2[x,y,z],{x,y,z}],{x,y,z}],Div|Grad];

Subscript[Γ2,O]=DirichletCondition[u2[x,y,z]==Exp[β*U[rOuterBoundary,0,0]],Norm[{x,y,z}]≥rOut
(*Subscript[Γ2,A]=DirichletCondition[u2[x,y,z]==0,Norm[{x,y,z}-reactionCenterCoord]≤rReaction

Subscript[Γ2,N]=NeumannValue[-κ*u2[x,y,z],

If[Norm[{x,y,z}-reactionCenterCoord]≤rReactionCenter,
 If[MemberQ[activesitecoorlist,{x,y,z}],True,False],
 False]

];


ufun=NDSolveValue[{op2==Subscript[Γ2,N],Subscript[Γ2,O]},u2,{x,y,z}∈mesh,Method→"FiniteElemen

density=Function[{x,y,z},ufun[x,y,z]*Exp[-β*U[x,y,z]]];
grad[x_,y_,z_]=Grad[ufun[x,y,z],{x,y,z}];
flux=Function[{x,y,z},-Exp[-β*U[x,y,z]]*grad[x,y,z]];

ActiveSiteArea=0;
ActiveSiteCoor={};
ActiveSiteNormal={};
Kon=0;
count=0;

Do[
 If[Norm[coordinatePairs[[i]][[1]]-reactionCenterCoord]≤ rReactionCenter,ActiveSiteArea+=Area
 Kon+=-flux[coordinatePairs[[i]][[1]][[1]],coordinatePairs[[i]][[1]][[2]],coordinatePairs[[i
 count+=1;
 ]

 ,
 {i,Length[coordinatePairs]}];

Kon*=ActiveSiteArea/count;

{density, flux,Kon}
]
```

Explanation :

solveSMOL[{bindingSiteStrength, κ, rBSpotential, Bcenter, finalmesh}]

bindingSiteStrength : value of Gb in kcal/mol

κ : intrinsic bimolecular rate constant, 1000000000000000 is sufficiently large to be considered as infinite

rBSpotential : radius of alternative binding site (unit : Angstrom)

Bcenter : coordinate of center of binding site

finalmesh : mesh of the protein model processed in MESH GENERATION

Output : {density, flux, bindingSiteStrength, Kon}

density : interpolating function of density.
Input : 3 D coordinate
Output : value of concentration of ligand at that coordinate.

flux : interpolating vector function of flux.
Input : 3 D coordinate .
Output : vector (3 values) of flux of ligand at that coordinate.

Kon : value of reaction rate divided by diffusion coefficient

Example : see below

```
input =
  Table[{i, 1000000000000000, 2, reactionCenterCoord + {3, 0, 0}, mesh}, {i, -5, 0}];
AbsoluteTiming[results = Map[solveSMOL, input]][[1]]
Table[{input[[i]][[1]], results[[i]][[-1]]}, {i, 6}]
```

```
942.727
```

```
{{-5, 2.57772}, {-4, 2.54821}, {-3, 2.51497},
 {-2, 2.45953}, {-1, 2.34409}, {0, 2.17475}}
```
