# Supplementary figures and images for: Structural and dynamic basis of substrate permissiveness in hydroxycinnamoyltransferase (HCT)
Source: PLoS Comput Biol. 2018 Oct 26;14(10):e1006511. doi: 10.1371/journal.pcbi.1006511 (PMC6203249; doi:10.1371/journal.pcbi.1006511)

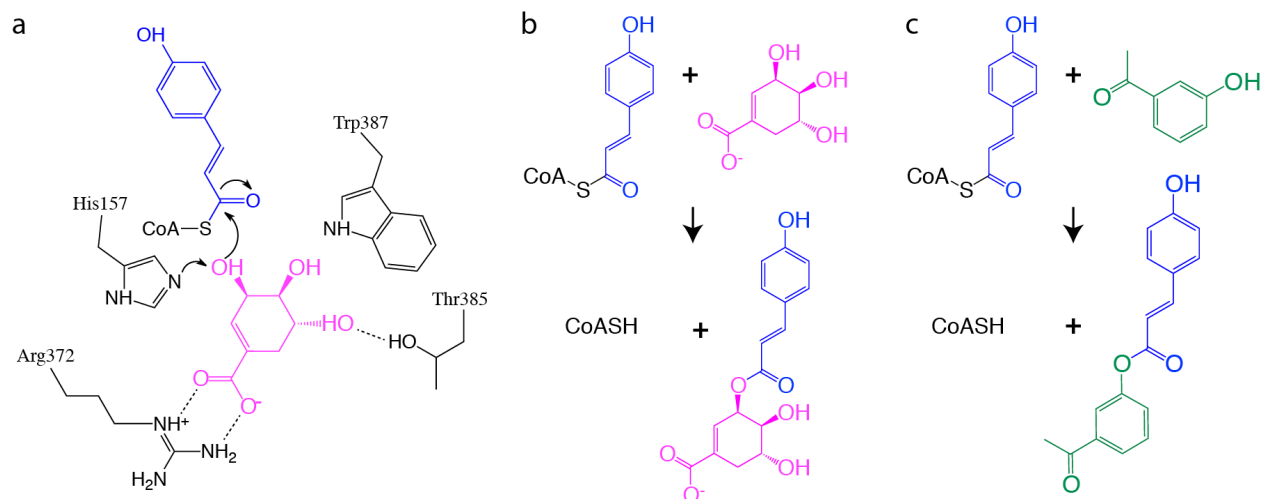

**S1 Figure**

Supplement: S1 Fig — (a,b) The p-coumaroyl group is transferred from p-coumaroyl-CoA to the native substrate shikimate, resulting in the product p-coumaroylshikimate. Residues are numbered according to SmHCT. (c) The acyl transfer reaction occurs between p-coumaroyl-CoA and 3-hydroxyacetophenone. (PDF) [file pcbi.1006511.s003.pdf]

a

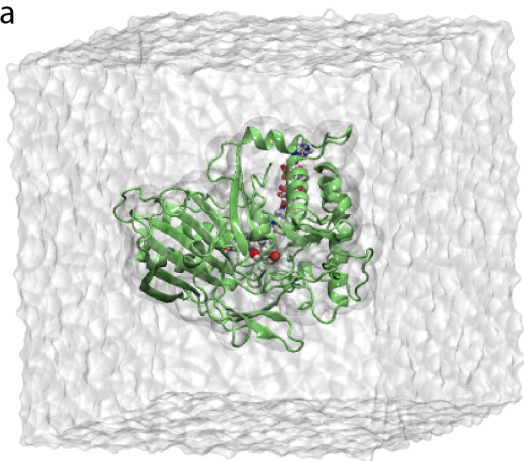

b

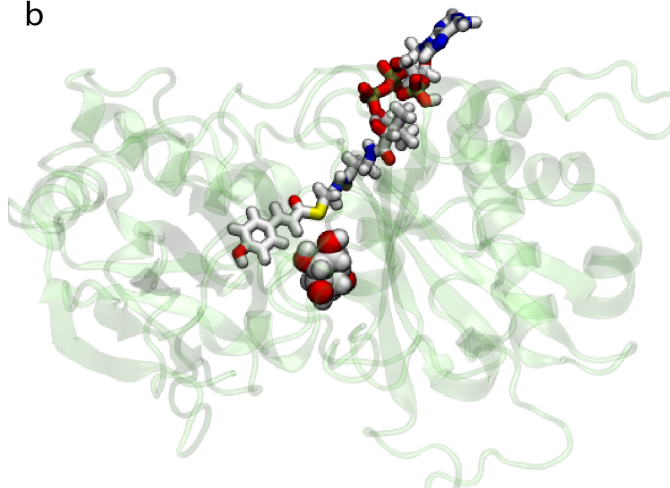

**S2 Figure**

Supplement: S2 Fig — The water box is shown as a transparent surface in (a) and the positions of p-coumaroyl-CoA (stick representation) and shikimate (vdW representation) are highlighted in (b). (PDF) [file pcbi.1006511.s004.pdf]

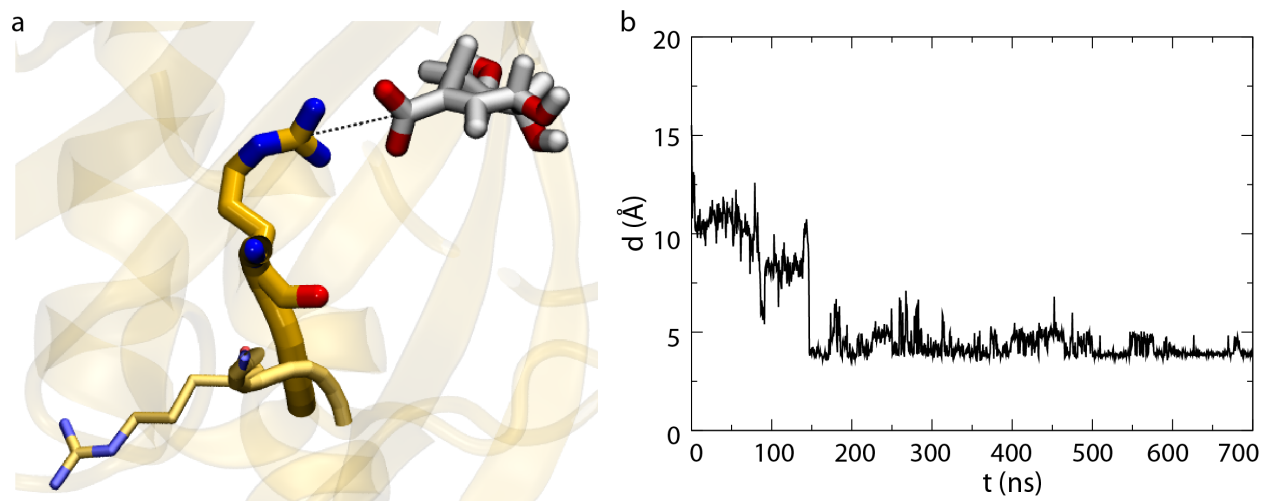

**S3 Figure**

Supplement: S3 Fig — Shikimate was placed within the SmHCT active site manually at the beginning of the simulation. (a) Snapshot of the system at t = 147 ns. The position of Arg372 at t = 0 ns is shown in thin sticks for reference. (b) Distance between the central carbon of the guanidinium group in Arg372 and the carbon of the carboxyl group in shikimate. (PDF) [file pcbi.1006511.s005.pdf]

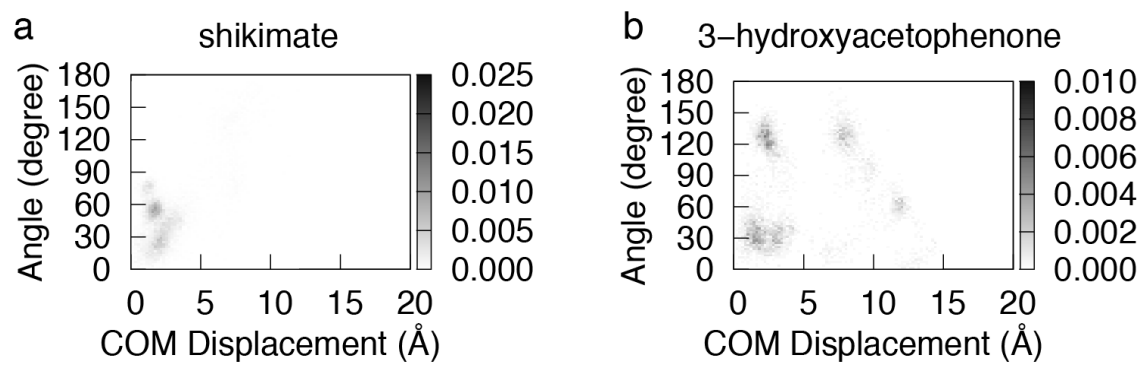

**S4 Figure**

Supplement: S4 Fig — Both the orientation and COM displacement are with respect to the CbHCT crystal structure. Calculations details as described in our previous work [10]. (PDF) [file pcbi.1006511.s006.pdf]

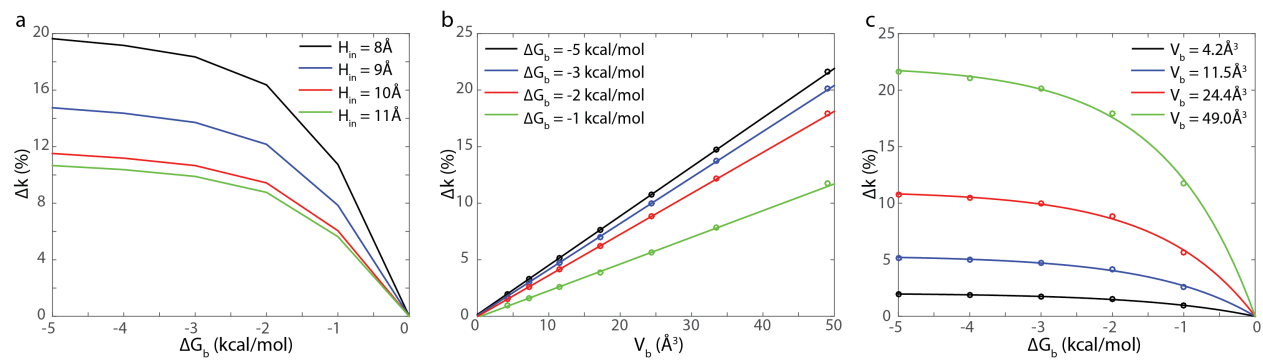

**S5 Figure**

Supplement: S5 Fig — (a) Δk decreases as the cylindrical enzyme elongates. The volume of the cylinder was kept approximately constant. The calculations were performed with rb = 2 Å, d = 3 Å and α = ∞. (b-c) With other metrics held constant, Δk scales linearly with the volume (Vb) of the off-center site (b), and has an exponential dependence on the binding affinity (ΔGb) of the site (c). The dots represent calculation results obtained with Hin = 9 Å, rin = 6 Å, d = 3 Å and α = ∞. The curves represent fitting results obtained with a linear (b) and an exponential (c) function, respectively. (PDF) [file pcbi.1006511.s007.pdf]

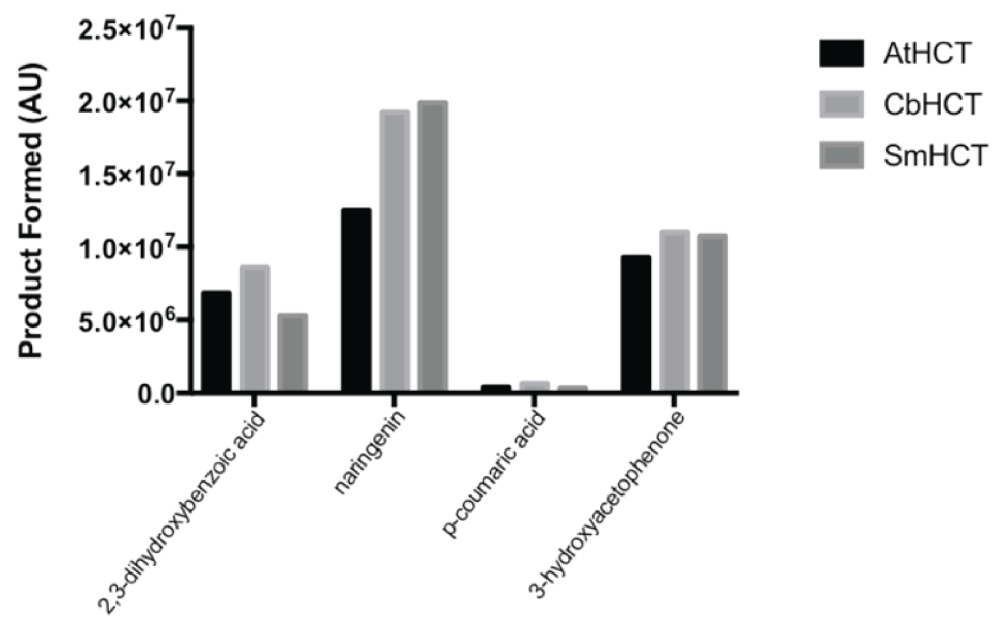

**S6 Figure**

Supplement: S6 Fig — Reactions were incubated overnight, and product formation measured via liquid chromatography-mass spectrometry. Reaction conditions as previously described [10]. (PDF) [file pcbi.1006511.s008.pdf]
