# Supplementary material for: Structural and dynamic basis of substrate permissiveness in hydroxycinnamoyltransferase (HCT)
Source: PLoS Comput Biol. 2018 Oct 26;14(10):e1006511. doi: 10.1371/journal.pcbi.1006511 (PMC6203249; doi:10.1371/journal.pcbi.1006511)
Supplement: S1 Table — (PDF) [file pcbi.1006511.s009.pdf]

S1 Table

AtHCT	CbHCT	CcHCT	SbHCT	SmHCT
Arg356	Arg350	Arg357	Arg371	Arg372
His153	His153	His153	His162	His157
Thr369	Thr363	Thr370	Thr384	Thr385
Trp371	Trp365	Trp372	Trp386	Trp387
Glu202	Glu202	Glu202	Glu211	Glu206
