# Supplementary material for: Structural and dynamic basis of substrate permissiveness in hydroxycinnamoyltransferase (HCT)
Source: PLoS Comput Biol. 2018 Oct 26;14(10):e1006511. doi: 10.1371/journal.pcbi.1006511 (PMC6203249; doi:10.1371/journal.pcbi.1006511)
Supplement: S2 Table — (PDF) [file pcbi.1006511.s010.pdf]

**S2 Table**

| HCT | Ligand(s) | Time (ns) | Notes |
|---|---|---|---|
| AtHCT | - | 1012 | Apo simulation |
| CbHCT | - | 1009 | Apo simulation |
| CcHCT | - | 989 | Apo simulation |
| SbHCT | - | 970 | Apo simulation |
| SmHCT | - | 977 | Apo simulation |
| AtHCT | *p*-coumaroyl-CoA+shikimate | 975, 895 | Holo simulation |
| CbHCT | *p*-coumaroyl-CoA+shikimate | 1010, 1008 | Holo simulation |
| CcHCT | *p*-coumaroyl-CoA+shikimate | 988, 989 | Holo simulation |
| SbHCT | *p*-coumaroyl-CoA+shikimate | 970, 975 | Holo simulation |
| SmHCT | *p*-coumaroyl-CoA+shikimate | 978, 989 | Holo simulation |
| CbHCT | *p*-coumaroyl-CoA+3-HAP | 1009 | Non-native substrate |
| SmHCT | *p*-coumaroyl-CoA+shikimate | 1134 | Shikimate initially placed in water |
