# Supplementary material for: Structural and dynamic basis of substrate permissiveness in hydroxycinnamoyltransferase (HCT)
Source: PLoS Comput Biol. 2018 Oct 26;14(10):e1006511. doi: 10.1371/journal.pcbi.1006511 (PMC6203249; doi:10.1371/journal.pcbi.1006511)
Supplement: S3 Table — (PDF) [file pcbi.1006511.s011.pdf]

S3 Table

HCT	Voume (Å^3)
AtHCT	2259
CbHCT	1673
CcHCT	1367
SbHCT	1460
SmHCT	3415
