# Supplementary material for: Structural and dynamic basis of substrate permissiveness in hydroxycinnamoyltransferase (HCT)
Source: PLoS Comput Biol. 2018 Oct 26;14(10):e1006511. doi: 10.1371/journal.pcbi.1006511 (PMC6203249; doi:10.1371/journal.pcbi.1006511)
Supplement: S4 Table — (PDF) [file pcbi.1006511.s012.pdf]

S4 Table

**Parameter**	**SmHCT**		
**PDB ID**	**6DD2**		
**Data Collection**			
Resolution range (Å)	61.54 - 2.81		
R_{merge}	0.141		
Total observations	61522		
Unique observations	15752		
Completeness (%)	98.3		
Multiplicity	3.9		
**Refinement**			
R_{work}	0.2480		
R_{free}	0.3084		
Resolution range	42.502 - 2.906		
Space group	P 21 21 21		
Unit cell (Å, ^{o})	79.390	83.749	188.855
	90.00	90.00	90.00
r.m.s.d. bonds (Å)	0.004		
r.m.s.d. angles (^{o})	0.725		
Ramachandran favored (%)	89.52		
Ramachandran outliers (%)	1.11		
Wilson B	46		
Protein and ligand atoms	6474		
Water atoms	0		
